# Intein-mediated site-specific conjugation of Quantum Dots to proteins *in vivo*

**DOI:** 10.1186/1477-3155-7-9

**Published:** 2009-12-10

**Authors:** Anna Charalambous, Maria Andreou, Paris A Skourides

**Affiliations:** 1 Department of Biological Sciences, University of Cyprus, P.O. Box 20537, 1678 Nicosia, Cyprus

## Abstract

We describe an intein based method to site-specifically conjugate Quantum Dots (QDs) to target proteins *in vivo*. This approach allows the covalent conjugation of any nanostructure and/or nanodevice to any protein and thus the targeting of such material to any intracellular compartment or signalling complex within the cells of the developing embryo. We genetically fused a pleckstrin-homology (PH) domain with the N-terminus half of a split intein (I_N_). The C-terminus half (I_C_) of the intein was conjugated to QDs *in vitro*. I_C_-QD's and RNA encoding PH-I_N _were microinjected into Xenopus embryos. *In vivo *intein-splicing resulted in fully functional QD-PH conjugates that could be monitored in real time within live embryos. Use of Near Infra Red (NIR)-emitting QDs allowed monitoring of QD-conjugates within the embryo at depths where EGFP is undetectable demonstrating the advantages of QD's for this type of experiment. In conclusion, we have developed a novel *in vivo *methodology for the site-specific conjugation of QD's and other artificial structures to target proteins in different intracellular compartments and signaling complexes.

## Background

The ability to target proteins *in *vivo with nanostructures and/or nanodevices is crucial both for understanding and controlling their biological function. Quantum Dots (QD's) serve as an ideal model nanostructure due to i) their superior optical properties that permit visual confirmation of *in vivo *targeting and localization and ii) their potential as a bio-imaging tool. In contrast to traditional fluorophores, QD's act as robust, broadly tunable nanoemitters that can be excited by a single light source, offer extremely high fluorescence intensity, wide excitation spectra, narrow and tunable emission spectra, large stokes shift and resistance to photobleaching [1]. Moreover, there is currently a limited number of FP's with emission in the Near Infra-Red (NIR) region. Despite claims of improved optical properties they are still far from optimal in terms of brightness and photostability, in comparison to NIR-QD's [2-4]. The NIR region of the spectrum (700-950 nm) is ideal for imaging through tissues because light scattering diminishes with increasing wavelength, and hemoglobin electronic and water vibrational overtone absorptions approach their minimum over this spectral domain. Furthermore living tissue auto fluorescence also reaches a minimum at this range and the fluorescent signal can, even in the case of organic fluorophores, be detected *in vivo *at subnanomolar quantities and at depths sufficient for experimental or clinical imaging [5]. The full potential of QD's is yet to be realized however because of limitations related to their relatively large size (typically 20-30 nm for biocompatible red-emitting QD's [1]), multivalency and the inability to genetically encode them. The first two issues have been resolved to a large extent with the synthesis of new types of QD's with much smaller hydrodynamic radii [6] and monovalent nanocrystals [7]. The third issue remains elusive and therefore addressed in this work using a simple intein-based method that allows the site-specific conjugation of QD's to any protein target *in vivo*, effectively overcoming the requirement to genetically encode QD's for tagging target proteins. In addition, this approach can be used to conjugate other nanostructures or nanodevices to target proteins and as a result to any intracellular compartment or protein signalling complex within the cell

Existing methods of QD-protein conjugation generally use either random chemical coupling with reactive amino-acids (e.g. -NH2, -COOH, -SH) on the protein surface or non-covalent complexation mediated by electrostatic interactions and ligand-recognition. A survey of site-specific bioconjugation methods led us to the intein-mediated ligation system. Inteins are polypeptide sequences that are able to self-excise, rejoining the two flanking extein sequences by a native peptide bond [8-10]. Inteins catalyze the splicing reaction through formation of an active thioester intermediate and have been widely used for *in vitro *protein semi-synthesis [9], segmental isotopic labelling [11] and *in vivo *protein cyclization [12]. This is the first time however that this approach has been used successfully in a vertebrate embryo to label proteins with QD's.

**Figure 1:**
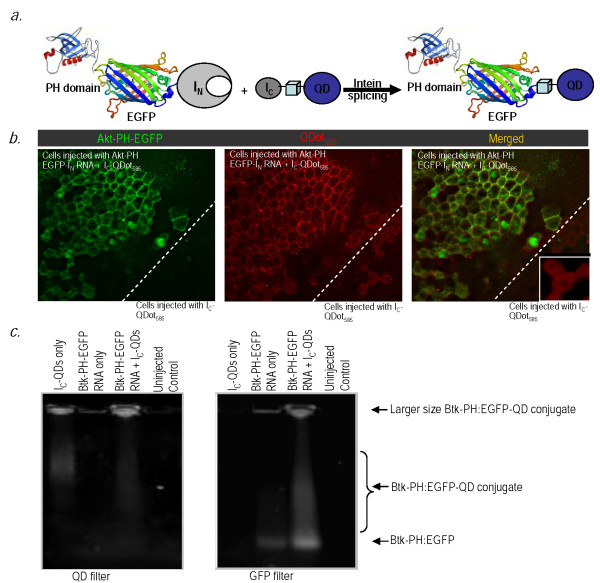
***In vivo *conjugation of QD's to Akt-PH-EGFP via intein mediated protein splicing**. **(a) **Schematic representation of site-specific intein-mediated conjugation of QD's to target protein. **(b) **Co-localization of QDot_585 _with Akt-PH-EGFP on the cell membrane. Fluorescence images of stage 10 Xenopus embryos microinjected with the probe (I_C_-QDot_585_) shown in red, in one blastomere at the two-cell stage, and then injected with RNA encoding the target protein (Akt PH-EGFP-I_N_) shown in green, in three of four blastomeres. Yellow shows the overlap between red QDot_585 _and green EGFP indicating successful QD-protein conjugation in a live embryo. **(c) **Biochemical characterization of protein-QD conjugates. Xenopus embryos were injected with either probe (Ic-QD's) only or Btk-PH-EGFP-I_N _RNA only or both, lysed at stage 10 and loaded onto a 0.5% agarose gel. QDot_655 _were visualized with a band pass 650/30 emission filter under UV excitation and GFP was imaged with a band pass 500/50 filter set on *UVP iBox *Imaging System. The ligation product appears as a smeary band under both the GFP and QD filters, only in lysates of Xenopus embryos injected with both the RNA and the probe, and is denoted as Btk-PH-EGFP-QD conjugate. A single band corresponding to the Btk-PH-EGFP protein fusion that is not conjugated to QD's is also detectable under the GFP filter, in lysates of Xenopus embryos injected with RNA only or RNA and probe, but not QD's only.

We selected to tag the PH domains of two proteins Akt and Btk. These were chosen due to their ability to translocate to the cell membrane upon PIP_3 _production by PI3-K [13] and would thus provide a clear visual confirmation of the conjugation in the intact embryo. Briefly, we genetically tagged EGFP fusions of the PH domains of Akt and Btk with the N-terminus half of a split intein (I_N_). The complementary C-terminus half of the intein (I_C_) was biotinylated and conjugated *in vitro *to streptavidin-coated QD's. The RNA's encoding Akt-PH-I_N _or Btk-PH-I_N _were delivered into Xenopus embryos via microinjection together with the I_C_-QD's. *In vivo *association of the intein halves in the cytosol triggered protein trans-splicing, resulting in the ligation of the QD to the target protein through a peptide bond (see Figure 1a).

We show in situ labeling of the PH domains of Akt and Btk with QD's using the above described intein-mediated ligation system. More specifically, we show that localization of the PH-QD conjugates can be monitored in real time in the developing Xenopus embryo. In addition we show that the QD tag does not affect the primary function of PH domains which is to recognize PIP_3_, as the ability to translocate from the cytosol to the plasma membrane is not compromised. Finally we show that in situ labeling of proteins with QDs offers significant advantages over labeling with traditional fluorophores and organic dyes.

## Materials and methods

### Embryos and explants

*Xenopus laevis *embryos from induced spawning [14] were staged according to Nieuwkoop and Faber (1967). Operation techniques and buffer (MMR, Ubbels, 1983) have been described [14]. *Xenopus *embryos were fertilized *in vitro *and dejellied using 2% cysteine-HCl, pH 7.8, then maintained in 0.1 × Marcc's Modified Ringer's (0.1 × MMR). Microinjections were performed in 4% Ficoll in 0.33 × MMR. The embryos were injected with RNA and Intein (I_C_) peptide-QD conjugates at the 2 and 4-cell stage according to established protocols [15]. After injections the embryos were cultured in 4% Ficoll in 0.33 × MMR until stage 8 and then cultured in either 0.1 × MMR or 400 nM Wortmannin (for some experiments) at room temperature. For *in vivo *assays, the embryos were transferred to slides for time lapse movies using Zeiss Axiocam MR3 and the Axiovision software 4.6 to monitor GFP-QD co-localization. For biochemical assays embryos were lysed and loaded onto agarose gels.

### Chemical Synthesis of biotinylated Intein (I_C_) peptide (I_C_-Biotin)

H-MVKVIGRRSLGVQRIFDIGLPQDHNFLLANGAIAANCFDYKDDDDK(Ahx-Biotin)G-NH2 Modifications: Biotin conjugated to lysine via a Ahx linker (6 carbon inert linker) A 47 amino acid peptide corresponding to C-terminal intein (I_C_) was synthesized on a 0.5 mmol scale on a 4-methylbenzhydrylamine (MBHA) resin according to the in-situ neutralization/HBTU activation protocol for Boc SPPS [16]. In order to put a biotin at C-terminus, it was necessary to add an extra amino acid, Lys, at the C-terminus. This Lys serves as a linking point for biotin as well as a spacer between the peptide and biotin. The peptide contains a cysteine protected with the NPyS group which was added as the last amino acid in the synthesis. Following chain assembly, global de-protection and cleavage from the support was achieved by treatment with HF containing 4% v/v pcresol, for 1 hour at 0°C. Following removal of the HF, the crude peptide product was precipitated and washed with anhydrous cold Et2O before being dissolved in aqueous acetonitrile (50% B) and lyophilized. The crude peptide was purified by preparative HPLC using a linear gradient of 25-45% B over 60 minutes. The purified peptide was characterized as the desired product by ESMS. The lyophilized peptide was dissolved in 60% DMSO at a concentration of 1 mg/ml.

### *In vitro *conjugation of I_C_-Biotin to streptavidin-coated QDs

I_C_-Biotin was diluted to 50 μM and used at 1:1 volume ratio with streptavidin-coated QDs (1 μM) (from Invitrogen or eBiosciences). To allow formation of the biotin-streptavidin bond we incubate at 24°C for 30 min. To remove any excess unbound peptide the conjugate was filtered through microcon centrifugal filter units (YM100) (Cat# 42413).

### Analysis of QD-peptide conjugates

Analysis of QD-peptide conjugation was performed by electrophoresis at 60 V for 4 h at 4°C using a 0.5% agarose gel. No loading buffer was added to the samples before loading. Gels were visualized under the ethidium bromide filter (515-570 nm) with a UVP Imager (data not shown).

Alternatively analysis of QD peptide conjugation was performed by spotting nitrocellulose membranes (Whatman). Biotinylated I_C _peptide and I_C _peptide that did not contain the biotin modification at the N-terminus were spotted on nitrocellulose membrane and blocked in PBS containing 1% BSA for 30 min at room temperature. The nitrocellulose membrane was then soaked in PBS containing streptavidin-coated QDs (1:500 dilution) for 30 min at room temperature. The membrane was washed with PBS-Tween 20 (1%) twice and visualized under the ethidium bromide filter (515-570 nm) with a UVP Imager (data not shown).

### Plasmids and Cloning

All plasmids were constructed using standard molecular biology techniques and they were sequenced to verify correct coding.

#### pCS2++-Btk PH-EGFP-I_N_

A PCR fragment amplified with IGpr62 (TGTACAGGCGCGCGTACGGCGGCGGCGGCGGCGGCAAGTTTGCGGAATA TTGCCTCAG) and IGpr64 (CGCGCGGCGGCCGCTTATTTAATTGTCCCAGCG) encoding I_N _with 5 N-terminal extein residues (KFAEY), using the pJJDuet30 plasmid (from Addgene) as template was inserted at the C-terminus of Btk-PH-EGFP [13] on pEGFPN1 between the BsrG I and Not I restriction sites. Btk-PH-EGFP-I_N _was then inserted into the multiple cloning site of the pCS2++ plasmid by restriction enzyme digest with EcoR I-Not I

#### pCS2++-Akt PH-EGFP-I_N_

A PCR fragment amplified with Apr1 (AAGATCGATATGAGCGACGTGGCTATTG) and Apr3 (AAGGAATTCCTTGTACAGCTCGTCCATGCCGAG) encoding Akt PH-EGFP, using the pAkt PH-EGFP-N1 plasmid [13] as template, was inserted into the multiple cloning site of the pCS2++ plasmid between the ClaI-EcoRI restriction sites. A PCR fragment amplified with IGpr61 (AAGGAATTCAAGTTTGCGGAATATTGCCTCAGTTTTGG) and IGpr63 (AAGCTCGAGTTATTTAATTGTCCCAGCG) encoding I_N _with 5 N-terminal extein residues (KFAEY), using the pJJDuet30 plasmid (from Addgene) as template was inserted at the C-terminus of Akt PH-EGFP on pCS2++ between the EcoRI-XhoI restriction sites.

All plasmids were transcribed into RNA using mMessage mMachine Sp6 kit (Ambion) and the mRNAs were purified using the Mega Clear kit (Ambion). Microinjections performed in Ficoll as mentioned above.

### Electrophoretic analysis of protein trans-splicing

Biochemical analysis of protein-trans splicing was performed by lysis of injected Xenopus embryos at stage 10. Lysis was performed by pipetting up and down in the presence of proteinase inhibitors (Sigma) and DNAse (Roche). Lysates were then loaded onto agarose gels run at 100 V for 2 h, at 4°C. Gels were visualized with a UVP Imager.

## Results and Disussions

To demonstrate in situ labeling of the target protein with QD's we injected both blastomeres of two-cell stage Xenopus embryos with the probe (I_C_-QDot_585_), allowed the embryo to develop to the four cell stage and then injected three out of four blastomeres with RNA encoding the target protein (in this case, Akt PH-EGFP-I_N_). The presence of EGFP on the PH domain allowed us to monitor and compare the distribution of the QD's vs the Akt-PH. As shown in Figure 1b, QD's translocated to the membrane in cells derived from the blastomere injected with both I_C_-QDot_585 _and RNA, where they colocalized with Akt-PH-EGFP. On the other hand, in cells that do not express the Akt-PH-EGFP-I_N_, QD's remained in the cytosol (see Figure 1b, third pane inset, at 20 × magnification). In addition cells in which the Akt PH-EGFP remained cytosolic, the QD conjugates also remained in the cytosol. To further establish that QD's were successfully conjugated to Akt-PH-EGFP *in vivo *we used a biochemical approach. Xenopus embryos injected as described above were lysed when they reached stage 10 and loaded onto an agarose gel. QDot_655 _were visualized with a band pass 650/30 emission filter under UV excitation and GFP was imaged with a band pass 500/50 filter set on *UVP iBox *Imaging System http://www.uvp.com/ibox.html. As shown in Figure 1c a single band of the expected molecular weight for the Btk-PH GFP appeared in lysates of Xenopus embryos injected with the RNA encoding the target protein (Btk PH-GFP-I_N_). This band could not be detected in lysates of Xenopus embryos injected with the probe (I_C _peptide conjugated QD_655_) only. A higher MW smeary band corresponding to the semi-synthetic product appeared only in lysates of Xenopus embryos injected with both the RNA encoding the target protein (Btk PH-GFP-I_N_) and the probe (I_C _peptide conjugated QD_655_). Importantly, this new band overlaps with the QD signal. The smeary appearance of the band in the agarose gel is due to the fact that the size of the protein-QD conjugates varies greatly as a result of the multivalency of commercially available streptavidin-coated QDs (4-10 streptavidin molecules (53 kD each)/QD giving 16-40 biotin binding sites implying 16-40 conjugated PH-GFP protein molecules per QD) resulting in a significant increase in size. In fact, the large size of some of the protein-QD conjugates combined with their lack of charge prevents them from migrating in the gels as they can be detected in the gel wells.

**Figure 2:**
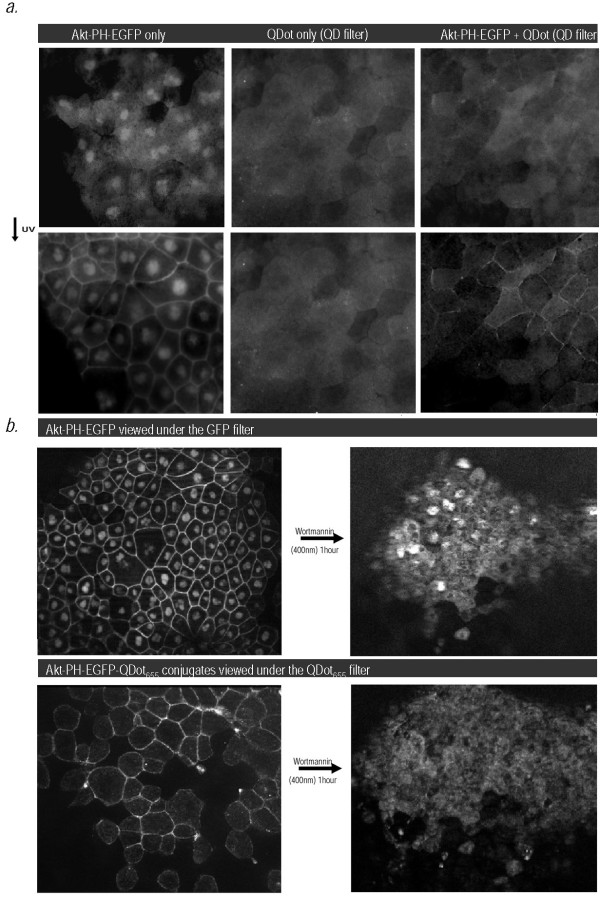
**UV-inducible and wortmannin-sensitive translocation of QD-Akt-PH-EGFP conjugates to the membrane**. (**a**) Akt-PH-EGFP protein fusions and Akt-PH-QDot_585 _conjugates translocate to the cell membrane upon exposure of injected Xenopus embryos to UV radiation. Live Xenopus embryos injected as described were imaged on a Zeiss Axioimager to visualize the localization of Akt-PH-EGFP and Akt-PH-QD conjugate before and after exposure to UV radiation for 5 min. Both Akt-PH-EGFP and Akt-PH-QD conjugates translocate to the cell membrane following brief exposure to UV radiation. **(b) **Translocation of Akt-PH-QD conjugates (QDot_655_) to the cell membrane is Wortmanin sensitive. Live Xenopus embryos were imaged on a Zeiss Axioimager to visualize the localization of Akt-PH-QD conjugate before and after treatment with Wortmannin (400 nM), a PI3-K inhibitor, for 1 hour. The Akt-PH QD conjugates become diffusely localized in the cytosol after treatemnt.

Previous studies have reported that UVB irradiation induces Akt activation and consequent translocation to the plasma membrane via a PI-3K/PDK dependent pathway as well as via Erks and p38 kinase through their downstream kinase, mitogen and stress-activated protein kinase Msk1 [17,18]. In agreement with these studies, upon exposure to UV light both the Akt-PH-EGFP and the QD conjugates translocated to the cell membrane within minutes, suggesting UV induced activation of PI3-K (Figure 2a). Furthermore the translocation of Akt-PH-EGFP and the QD conjugates to the plasma membrane was completely eliminated by wortmannin, a PI3-K specific inhibitor suggesting that the observed translocation is PI3-K dependent (Figure 2b). Collectively the data in Figures 1 and 2 show that the QD's were a) successfully conjugated to Akt-PH-EGFP *in vivo *and b) the QD tag did not affect the primary function of the PH domain, which is to recognize PIP_3 _and translocate to the cell membrane.

**Figure 3:**
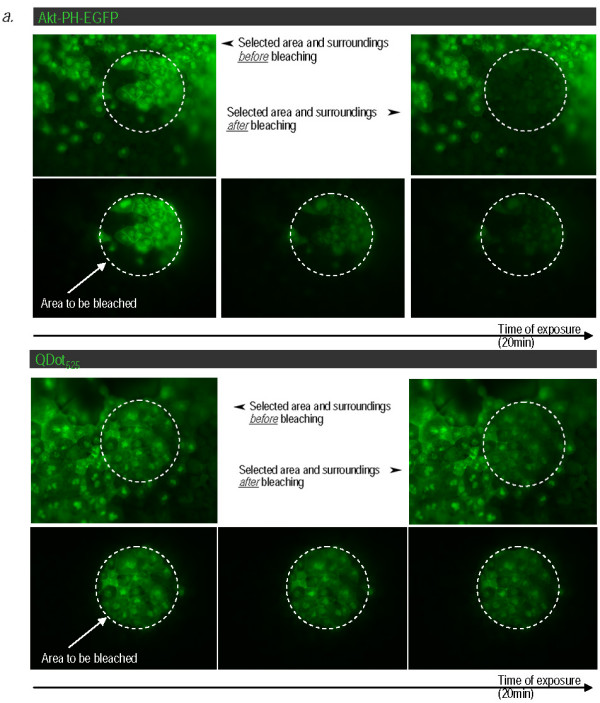
**QD-Akt-PH conjugates are resistant to photobleaching, unlike Akt-PH-EGFP fusions**. Fluorescence images of stage 10 Xenopus embryos microinjected with the probe (I_C_-QD_525_) and with RNA encoding the target protein (Akt PH-EGFP-I_N_), both shown in green since their emission spectra are closely matched. Embryos were exposed to continuous excitation (~ 480 nm) for > 20 min. This led to gradual loss of the EGFP signal but did not affect the QDot_525 _signal.

We went on to compare the photostability of the QD-conjugates to that of EGFP. To test this we used the QDot_525_-Streptavidin from Invitrogen which have emission spectra that closely match those of EGFP and repeated the conjugation and injections as described above. It should be noted that unlike the QDot_585 _Streptavidin conjugates which fail to enter the nucleus, QDot_525_-Streptavidin have sufficiently small hydrodynamic radii to do so. Injected embryos were allowed to develop to stage 10 and were then imaged on an epifluorescence microscope. Figure 3 shows that continuous exposure of the embryos to excitation light (~ 480 nm) led to gradual loss of the EGFP signal, due to photobleaching, but did not affect the QDot_525_-Streptavidin signal even after 20 minutes of continuous excitation. Importantly and despite the long exposure to excitation light the QD conjugates retained their membrane localization.

**Figure 4:**
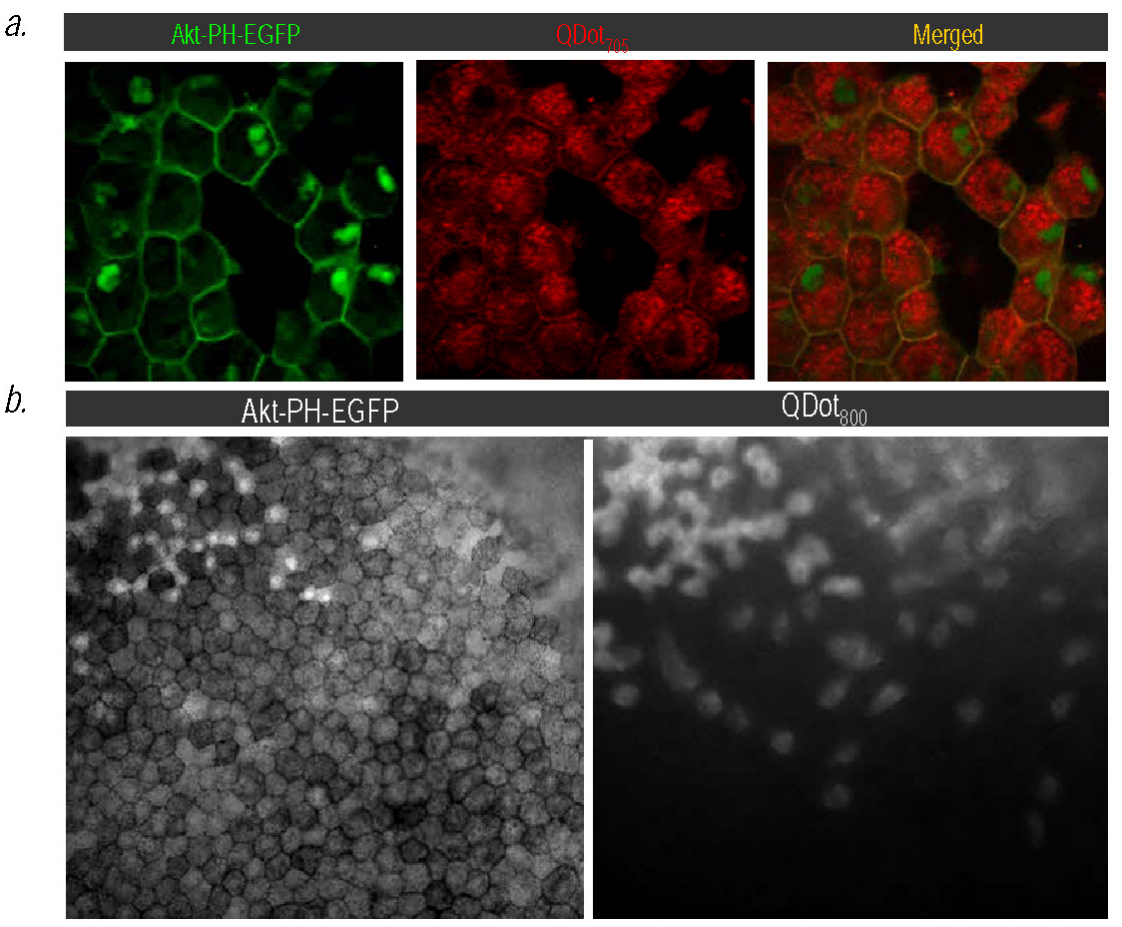
**Increased NIR-QDot size imposes constraints on Akt-PH-QD conjugate translocation efficiency but NIR-QD's allow visualization in deeper cell layers in a live Xenopus embryo, unlike Akt-PH-EGFP (a) Co-localization of QDot_705 _with Akt-PH-EGFP on the cell membrane**. Note that unlike the QDot_585_, the QDot_705 _are not recruited as effectively to the cell membrane. **(b) **QDot_800 _allow visualization of the Akt-PH ~ two to three layers below the superficial cell layer, where the GFP signal was either undetectable or too diffuse. The images are of the same region of the embryo imaged with a GFP (left) and a QDot_800 _(right) filter set.

The possibility of taking advantage of the NIR region of the spectrum, which is ideally suited for biological imaging [19] was one of the reasons we developed this system. We have recently shown that labelling of blastomeres with NIR QD's enables visualization of deep tissue movements with single cell resolution [20]. We postulated that NIR QD labelling of a protein would enable the visualization of protein localization in the living embryo beyond the superficial cell layers. To achieve this we used streptavidin-coated NIR QD's (emission maxima centered at 800 nm). NIR QDot_800 _enabled the visualization of the Akt-PH several cell layers deep where the GFP signal is either undetectable or too diffuse to provide any meaningful information (see Figure 4b).

Despite their ideal optical properties, commercially available CdSe/ZnSe QD's especially those emitting in the NIR are large and can impair trafficking of proteins to which they are attached and limit access to crowded cellular locations such as the cell membrane or even restrict access into membrane bound intracellular compartments such as the nucleus [7,20,21]. A large fraction of the QD size comes from the passivating layer, often a polyacrylic acid polymer or phospholipids micelle, required to allow conjugation of biological molecules to QD's and retention of their optical properties [1]. We used commercially available QD's from Invitrogen and eBiosciences (15-20 nm in diameter), that did not only have the passivating layer but were further coupled to streptavidin, as described earlier. Conjugates of Akt-PH with QD's from all the emission wavelengths tested (525, 565, 585, 605, 705, 800) could translocate to the cell membrane, However, there was a definitive size dependence in their ability to do so, with longer wavelength emitting QD's showing a diminished capacity to do so (compare Figure 3 (QDot_525_) to Figures 1b (QDot_585_) and Figure 4a (QDot_705_). In addition and in agreement with previously published work [20], only protein conjugates with QDot_525 _(from Invitrogen) and QD_605 _(from eBiosciences), were able to translocate into the nucleus, as efficiently as an organic fluorophore conjugate (Cy3) (compare Figure 3 to Figure 1b (QDot_585_), Figure 4a (QDot_705_) and Figure 2b (QDot_655_) and data not shown). Longer wavelength protein-QD conjugates were completely excluded from this membrane bound intracellular compartment. The fact that QD's_605 _from eBiosciences but not from Invitrogen entered the nucleus could be a result of different coating of the QD's or lower number of streptavidin molecules per nanocrystal. Our present results point to the need for wider availability and commercialization of significantly smaller water soluble nanocrystals with a variety of core and shell compositions as synthesized by different groups [6,22-24].

## Conclusion

Herein, we describe a simple and effective method that enables the site-specific conjugation of QD's and other artificial structures to target proteins *in vivo*. QD's were chosen as a model nanostructure due to their superior optical properties that facilitate detection and enable evaluation of the conjugation method. *S*ite-specific conjugation of QD's to proteins was afforded by intein-based protein trans-splicing. Unlike other conjugation methods, the intein method is a traceless ligation, that is the intein itself is spliced out and excluded from the final conjugation product. In addition to site-specificity, intein-based protein trans splicing has several other advantages, including high efficiency of product formation, reproducibility and versatility as it allows the targeting of any nanoparticle (QD or other) to a protein of interest.

An important feature of this conjugation method is the fact that target protein functionality is not affected upon fusion with QD's. In fact QD-PH conjugates retained full functionality of the PH domain as indicated by their ability to i) recognize PIP3, and ii) to translocate to the cell membrane in a PI3-K dependent manner (Figure 1b-d). This should hold true for most proteins as the QD is fused post-translationally to the target protein and does not therefore influence protein folding and tertiary structure, in contrast to fluorescent protein fusions.

In addition, conjugation of QD's to the PH domain did not affect the ability of the former to resist photodegradation. Photostability is one of the main advantages of QD detection as it allows prolonged visualization of the labelled protein and thus facilitates determination of its function as well as delineation of the pathway in which it is involved. We found no loss of fluorescence intensity in PH-QD conjugate injected embryos even after 20 min of continuous illumination, whereas there was complete loss of EGFP fluorescence after 5 min of illumination (Figure 2).

Moreover, this conjugation method is easily adaptable to the needs of the individual experiment as it allows use of different streptavidin-coated QD's (emitting at different wavelengths) to observe the same target protein, without having to change any other reagent in the experiment. For instance, use of Near Infra Red (NIR)-emitting QDs allowed monitoring of QD-conjugates within the embryo at depths where EGFP is undetectable demonstrating the advantages of NIR-QD's for this type of experiment.

However, our present results point to the need for wider availability and commercialization of smaller water soluble nanocrystals and controlled nanoparticle valency. The combination of efficient and non-reversible fusion of QD's to target proteins with reduced QD size and monovalency could help to make the strategy described in this paper a standard tool for in vivo imaging of protein dynamics at the single-molecule level. Finally, this methodology could be invaluable due to its potential diagnostic and therapeutic implications, as it makes the targeting of nanostructures and nanodevices to different intracellular compartments and signaling complexes a viable possibility.

## Competing interests

The authors declare that they have no competing interests. Please see accompanying declaration.

## Authors' contributions

PS conceived of the study, participated in its design and coordination and helped to draft the manuscript. AC participated in the design of the study, carried out the molecular and biochemical studies and drafted the manuscript. MA performed the in vivo assays. All authors read and approved the final manuscript.
